# Sub-Telomeric *core X* and *Y'* Elements in *S.cerevisiae* Suppress Extreme Variations in Gene Silencing

**DOI:** 10.1371/journal.pone.0017523

**Published:** 2011-03-17

**Authors:** Patricia Power, Daniel Jeffery, Muhammad Attiq Rehman, Arjun Chatterji, Krassimir Yankulov

**Affiliations:** Department of Molecular and Cellular Biology, University of Guelph, Guelph, Ontario, Canada; Texas A&M University, United States of America

## Abstract

Telomere Position Effect (TPE) is governed by strong repression signals emitted by telomeres via the Sir2/3/4 Histone Deacetylase complex. These signals are then relayed by weak proto-silencers residing in the subtelomeric *core X* and *Y'* elements. Subtelomeres also contain Sub-Telomeric Anti-silencing Regions (*STARs*). In this study we have prepared telomeres built of different combinations of *core X, Y'* and *STARs* and have analyzed them in strains lacking Histone-Acetyltransferase genes as well as in *cdc6-1* and *Δrif1* strains. We show that *core X* and *Y'* dramatically reduce both positive and negative variations in TPE, that are caused by these mutations. We also show that the deletion of Histone-Acetyltransferase genes reduce the silencing activity of an *ACS* proto-silencer, but also reduce the anti-silencing activity of a *STAR.* We postulate that *core X* and *Y'* act as epigenetic “cushioning” *cis*-elements.

## Introduction

Gene silencing refers to position dependent and promoter-independent repression of genes. It is characterized by local histone hypoacetylation and the formation of heterochromatin structures. In *S.cerevisiae,* gene silencing operates at the mating type loci *HML* and *HMR*, at the *rRNA* gene cluster and in the sub-telomeric regions of the chromosomes [1]. Gene silencing at subtelomeres is referred to as Telomere Position Effect (TPE) and is governed by strong repression signals emitted by the telomere itself [1]. These signals are relayed by weaker proto-silencers, which are positioned in the subtelomeric *core X-* and *Y'-* elements [2]. To date, proto-silencer activity has been assigned to *ARS* consensus sequences (*ACS*) and for the binding sites for Rap1p and Abf1p [3], [4], [5], [6]. The subtelomeres also contain sequences, which display anti-silencing properties and are referred to as *STARs* (Sub-Telomeric Anti-silencing Regions) [7]. The antagonizing silencing and anti-silencing activities emitted by these elements confer a peculiar quasi-unstable mode of subtelomeric gene expression. Any gene residing in the subtelomeres or translocated to these loci acquires either fully silenced or fully active state. This state is maintained through many generations, however infrequent switches occur to produce expression patterns that are reminiscent to the classical variegated pigmentation in the eye of Drosophila [8]. In all cases, the transition between the silenced and active states of expression is accompanied by histone acetylation and other post-translational histone modifications [1].

A Histone DeAcetylase (HDAC), Sir2p, plays a central role in the establishment and maintenance of silencing at all repressed loci. At telomeres there are two means of engaging Sir2p. The telomeric TG_1-3_ repeats bind Rap1p, which in turn recruits Sir3p and Sir4p to eventually recruit Sir2p [1]. Two proteins, Rif1p and Rif2p, interfere with the interaction between Rap1p and Sir3/Sir4 thus acting as anti-silencing factors [9], [10], [11]. At the same time the sub-telomeric *ACS* proto-silencers bind ORC (Origin Recognition Complex). *ACS*-bound Orc1 associates with Sir1p to independently recruit Sir2p to these positions [1]. Consequently, Sir2p deacetylates the nearby nucleosome and spreads over the neighboring ones with the aid of Sir3p and Sir4p. The spreading of histone deacetylation by Sir2p is counteracted by Histone Acetyl Transferases (HAT), but the mode of their action is not understood to the extent of the *SIR* genes.

HATs acetylate lysines of core histones to generate events, which culminate in chromatin de-condensation. To date, nine HATs have been described in *S.cerevisiae* [12]. Several studies have pointed to *SAS2* as the principal *SIR2*-counteracting HAT at telomeres [13], [14], [15], [16], [17]. Sas2p is responsible for the acetylation of H4-K16 *in vivo*, while Sir2p is deacetylating this position [14], [15]. Thus, the two opposing enzymes generate a dynamic chromatin boundary at subtelomeres. Paradoxically, deletion of *SAS2* very moderately increases the silencing of natural subtelomeric genes [14], [15], but dramatically reduces silencing at synthetic telomeres thus portraying *SAS2* as an anti-silencing factor [18], [19], [20], [21]. This stark discrepancy has not been adequately explained. On the other hand, many other lysines in H3 and H4 are hypo-acetylated in subtelomeric chromatin [22] suggesting that other HATs are also directly involved in anti-silencing.

In this study we have characterized the roles of five HATs *(HAT1, GCN5, SAS2, SAS3, Rtt109)*, of *RIF1* and *CDC6* on several recombinant telomeres build up of *core X, Y'* and *STARs.* These mutations produced both positive and negative effects on telomeric silencing. Unexpectedly, we have revealed that subtelomeric *core X* and *Y'* dampened down the extreme deviations of TPE caused by these mutations.

## Materials and Methods

### Yeast strains

Yeast strains with deletions of *HAT1, GCN5, SAS2, SAS3, YNG1, Rtt109* and *RIF1* are derivatives of *BY4742* and were obtained from ATCC. All other mutants are derivatives of *W303*. All strains used in this study are listed and referenced in Table 1.

**Table 1:** Yeast strains used in this study.

Strain	Genotype	Reference
*BY4742*	*his3Δ1 leu2Δ0 lys2Δ0 ura3Δ0 MATα*	
*Δsas2*	*BY4742 sas2::KanMX*	ATCC#4016568
*Δsas3*	*BY4742 sas3::KanMX*	ATCC#4013078
*Δyng1*	*BY4742 yng1::KanMX*	ATCC#4011840
*Δrtt109*	*BY4742 rtt109::KanMX*	ATCC#4011490
*Δhat1*	*BY4742 hat1::KanMX*	ATCC#4012827
*Δgcn5*	*BY4742 gcn5::KanMX*	ATCC#4017285
*Δrif1*	*BY4742 rif1::KanMX*	ATCC#4017170
*cdc6-1*	*cdc6-1 ade2-1 trp1-1 can1-100 leu2-3,112 his3-11,15 ura3-1 MAT* **a**	[38]
*orc2-1*	*orc2-1 ade2-1 trp1-1 can1-100 leu2-3,112 his3-11,15 ura3-1 MAT* **a**	[39]
*orc5-1*	*orc5-1 ade2-1 trp1-1 can1-100 leu2-3,112 his3-11,15 ura3-1 can1-100 MAT* **a**	[40]
*cdc45-1*	*cdc45-1 ade2-1 trp1-1 can1-100 leu2-3,112 his3-11,15 ura3-1 MAT* **a**	[41]
*scdc7-1*	*cdc7-1 ade2-1 trp1-1 can1-100 leu2-3,112 his3-11,15 ura3-1 MAT* **a**	[41]
*mcm5-461*	*mcm5-461 ura3-52 leu2-3,112 ade2 lys2-801 MATα*	[42]

### Telomeric constructs

**Figure 1:**
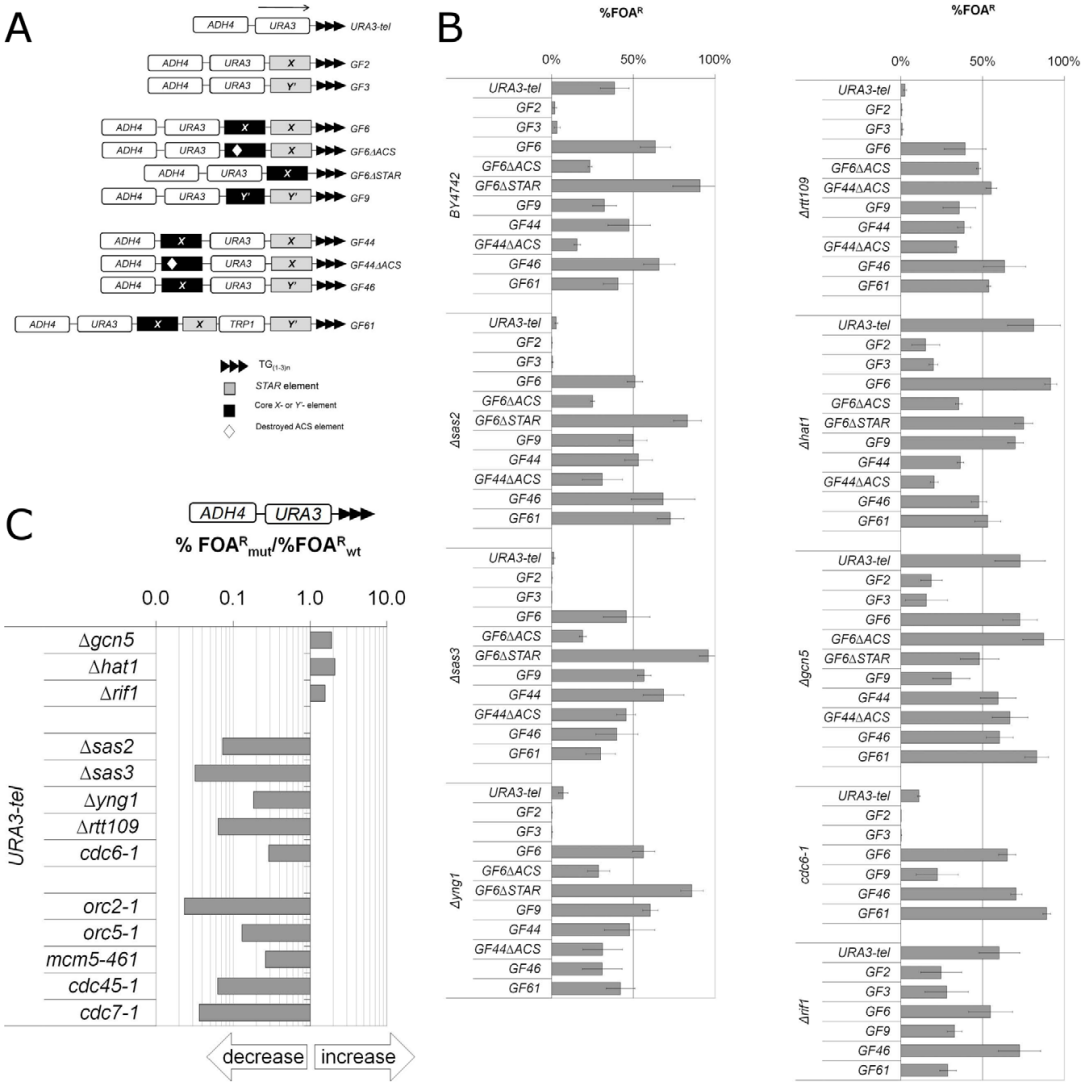
Analysis of Telomere Position Effect in Histone-Acetyl-Transferase Mutants. **A) Telomeric reporters used in this study.** Maps (not to scale) of the used constructs are shown. The positions of *core X* element from the *IIR* telomere and the *Y'* element from the *XII-L* telomere (black rectangles), the *STARs* from the same telomeres (grey rectangles), *URA3*, *ADH4* and the telomeric TG_1-3_ repeats (black triangles) are as indicated. The position of the destroyed *ACS* (*ARS* Consensus Sequence) is depicted by an open diamond. The 5′→3′ direction of *URA3* transcription is indicated in the URA3-tel construct (top) and is the same for all constructs shown. The insertions between *URA3* and the telomeric repeat add 145-900 base pairs as compared to URA3-tel. **B) Percentage of FOA^R^ cells in different strains and constructs.** The reporter constructs shown along the vertical axis were integrated in the strains shown on the left. Percentage of FOA^R^ cells was measured in at least three independent experiments. Average %FOA^R^ ± std. dev. were calculated and plotted. Data is from Table S1. **C) URA3-tel recapitulates silencing effects in mutant strains.** The URA3-tel construct was integrated in the strains shown along the vertical axis. The ratios of %FOA^R^ in the mutant strains versus the %FOA^R^ in the isogenic *wild type* strain were calculated and plotted. The effects of *Δsas2, Δsas3, Δyng1, Δrtt109, Δhat1*, *Δgcn5* and *Δrif1* were assessed using *BY4742* as the wild type strain (Table S1). The effects of *orc2-1, orc5-1, mcm5-461, cdc6-1, cdc45-1* and *cdc7-1(sas1)* were assessed using *W303* as the wild type strain (data not shown). There is little difference in the levels of telomeric silencing between *BY4742* and *W303*. The arrows underneath the exponential graph indicate increase or decrease of silencing.

All constructs are flanked by a portion of *ADH4* and telomeric TG_1-3_ repeats (see Fig. 1A) and are designed for targeted integration in the left telomere of chromosome *VII*. URA3-tel [23], GF2, GF3, GF6, GF9, GF44, GF46 and GF61 [4] were previously described. GF6ΔSTAR, GF6ΔACS and GF44ΔACS were produced by excision of the *STAR* element in GF6 or by site directed mutagenesis of *ACS* in the *core X* elements, respectively. All integrating constructs were produced by restriction digestion of the corresponding plasmids.

### Telomeric integration and analysis of gene silencing

Cells were transformed with integrating constructs and three single colonies were selected from SC-ura plates. To warrant for the loss of un-integrated constructs (linear DNAs lacking CEN elements), transformants were restreaked on Sc-ura and again a single colony from this SC-ura plate was streaked on both SC-ura and SC/FOA. Fluoro-orotic acid (FOA) has a selective toxicity for cells expressing *URA3*, hence SC/FOA selects for the repressed state of *URA3* and confirms variegated expression. By the tird re-streaking the transformed cells have been grown for about 60 generations. This procedure uniformly produces cells that have integrated the test constructs (Fig. 1) in the *VIIL* telomere when analyzed by PCR. Finally, a single colony was taken from the third SC-ura plate and grown for about 30 generations in non-selective (YPD) medium. Serial 1∶10 dilutions were prepared for each culture and 5 µl aliquots were spotted on SC and SC/FOA plates. Colonies in two consecutive spots with less than 50 colonies (these correspond to two consecutive dilutions) were counted. The %FOA^R^ for each independent culture was acquired as the number of colonies on SC/FOA plates divided by the number of colonies on SC plates. Finally, the average %FOA^R^ of the counts in three independent cultures ± standard deviation were calculated and are shown in Table S1. Average values and the ratios between %FOA^R^ in different strains and/or constructs were calculated and plotted in Microsoft Excel.

## Results

### *Core X* and *Y'* curtail variations in TPE caused by deletion of HAT genes

We used the set of telomeric reporters shown in Fig. 1A to analyze the role of several non-essential HATs in TPE. These reporters contain *URA3* and different combinations of subtelomeric *core X*, *Y'* and *STAR* elements (Fig. 1A). The ADH4-URA3-tel construct [23] is one of the most frequently used telomeric reporters and serves as a direct cross-reference between other studies and the current one. GF2 and GF3 contain *STARs* derived from the *core X-IIR* or *Y'-XIIL* elements, respectively. GF6 and GF9 contain the same *STARs,* but also the *core X* from the same telomeres, respectively. In GF44 and GF46 the *core X* and the *Y'* are positioned distal to the telomere beyond *URA3*. In GF61 *URA3* is away from the telomere beyond two *STARs*, *core X* and *TRP1*. In addition, *ACS* and *STAR* were destroyed in GF6 and GF44 as indicated. The insertions between *URA3* and the telomeric repeat add 145-900 base pairs in different constructs as compared to URA3-tel. Several studies have shown that the telomeric silencing for these and other constructs does not directly correlate to the distance from the telomeres [2], [3], [4], [24], [25]. Instead, silencing is discontinuous and is strongly influenced by the nature and the positions of different regulatory elements [2], [26]. Therefore, the variety of elements in these constructs allows for broad assessment of TPE in different strains.

All constructs were integrated in the left telomere of chromosome *VII* in *BY4742* and its derivatives *Δsas2, Δsas3, Δyng1, Δrtt109, Δhat1* and *Δgcn5* and selected on SC-ura plates. Colonies were then streaked on SC/FOA plates, which render the *URA3*-expressing cells sensitive to the drug while the cells with repressed *URA3* form FOA^R^ colonies. After confirming the variegated mode of expression of the integrated reporters, three colonies were grown in non-selective medium for 30 generations to allow for the re-establishment of the silenced/active equilibrium of *URA3* in these cultures. The percentage of FOA^R^ was calculated as the number of colonies on SC/FOA plates divided by the number of colonies on SC plates. The average values ± standard deviations were calculated (Table S1) and are plotted in Figure 1B.

Next, we cross-referenced the acquired data to available data in earlier publications. URA3-tel, GF2, GF3, GF6, GF9, GF44, GF46, GF61, GF6ΔSTAR, GF6ΔACS and GF44ΔACS showed very similar levels of %FOA^R^ in *BY4742* cells as compared to the previously used *W303* strain [4], [24], [25]. In addition, the prototype URA3-tel construct recapitulated the silencing defects observed in *sas2, sas3, orc2-1, orc5-1, mcm5-461, cdc6-1, cdc45-1* and *cdc7-1(sas1)* (Fig. 1C) [18], [19], [27], [28]. Finally, we compared the magnitude of *SAS2*-dependent de-repression of URA3-tel in *BY4742* and *W303* (the only available data for direct comparison that we are aware of). The deletion of *SAS2* in *W303* had decreased repression in the range of 10-50 fold [21], [29], while in *BY4742* we observed a reduction of 14 fold. Thus, our data is in close agreement with all earlier studies. We used the values in Table S1 to calculate the ratios of %FOA^R^ in the mutant strains versus the %FOA^R^ in the isogenic *wild type BY4742* strain. These ratios provide quantitative assessment of the effect of each gene on the silencing of *URA3* in each individual construct.

**Figure 2:**
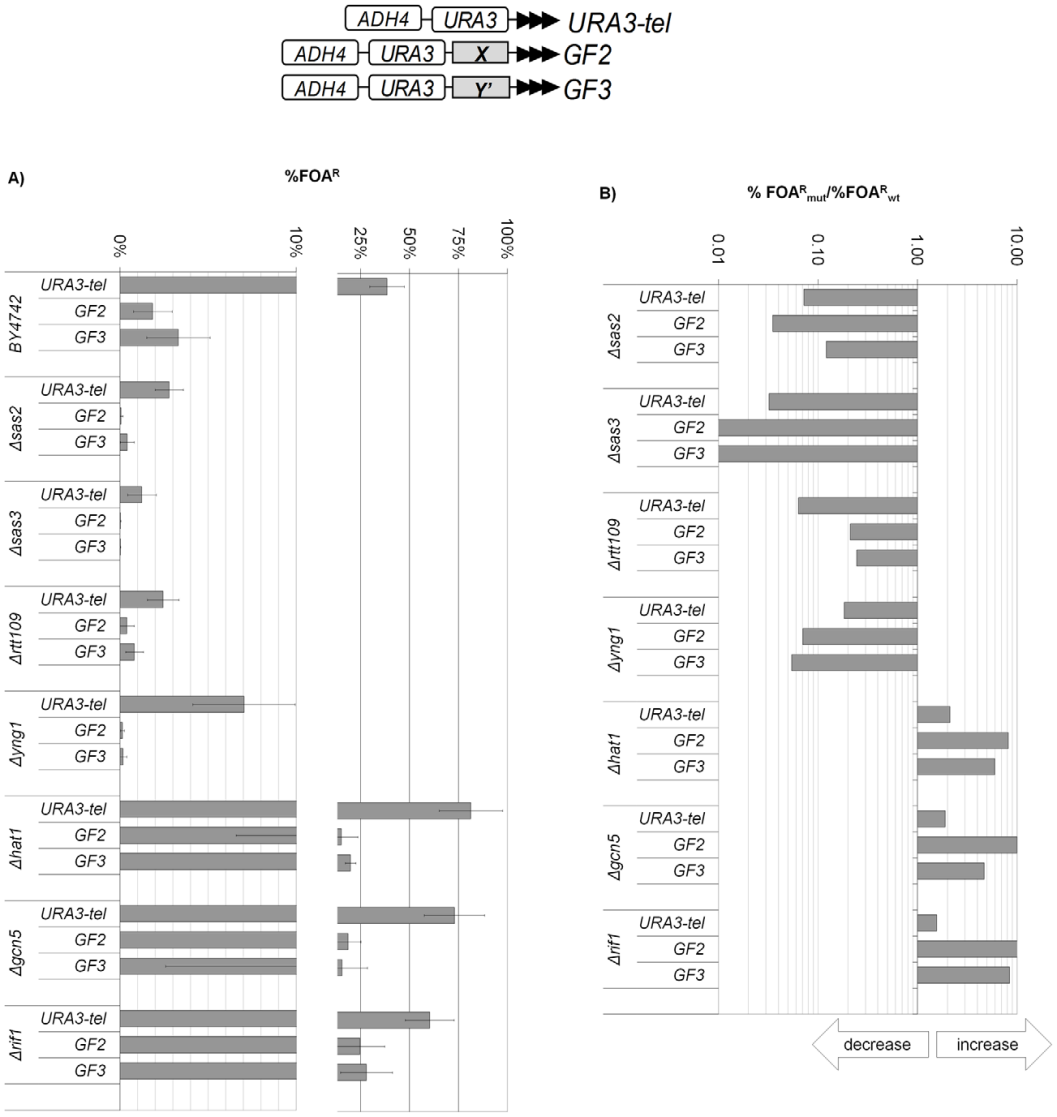
Alterations of TPE in constructs lacking *core X* or *Y'* elements. The URA3-tel, GF2 and GF3 constructs (shown on top) were integrated in the strains shown on the left and the level of *URA3* silencing was calculated as %FOA^R^ cells. **A) Levels of** ***URA3*** **silencing (%FOA^R^).** The 0-10% range is spread out to properly show differences at very low levels of silencing. **B) Ratios of %FOA^R^ in the mutant strains versus the %FOA^R^ in the** ***wild type*** **(** ***BY4742*** **) strain.** Data is from Table S1. The arrows underneath the exponential graph indicate increase or decrease of silencing.

The deletion of *SAS2* and *SAS3* caused 10-100 fold de-repression in URA3-tel, GF2 and GF3, whereas the deletion of *YNG1* ((a modulator of *SAS3* activity in the NuA3 complex [30]) and *Rtt109* caused 5-50 fold decrease of repression (Fig. 2B). In contrast, the deletion of *HAT1* and *GCN5* moderately (2-10 fold) increased repression (Fig. 2B). The gain in silencing in *Δhat1* and *Δgcn5* cells is comparable to the effect of the deletion of *RIF1* (Fig. 2B), a key telomeric anti-silencing factor. We do not understand the mechanisms that lead to these somewhat surprising effects for HAT genes. However, the similarity in the magnitude of effects in *Δhat1*, *Δgcn5* and *Δrif1* cells indicates that the increase in repression in *Δhat1* and *Δgcn5* is significant.

Hence, at telomeres lacking *core X* or *Y'* elements different HATs operate by different mechanisms and can produce both positive and negative effects on TPE. As expected, the addition of *STARs* in GF2 and GF3 further reduced the level of silencing in *Δsas2, Δsas3, rtt109* and *Δyng1* cells. Surprisingly, the calculations for *Δhat1* and *Δgcn5* cells showed that the addition of *STARs* generated modest, but consistent increase in telomeric silencing. It is conceivable that *STAR* activity is diminished in these mutants. Alternatively, the overall increase of telomeric silencing in them can over-compensate for the anti-silencing effect of *STARs*. We deal with this ambiguity in Fig. 6.

**Figure 3:**
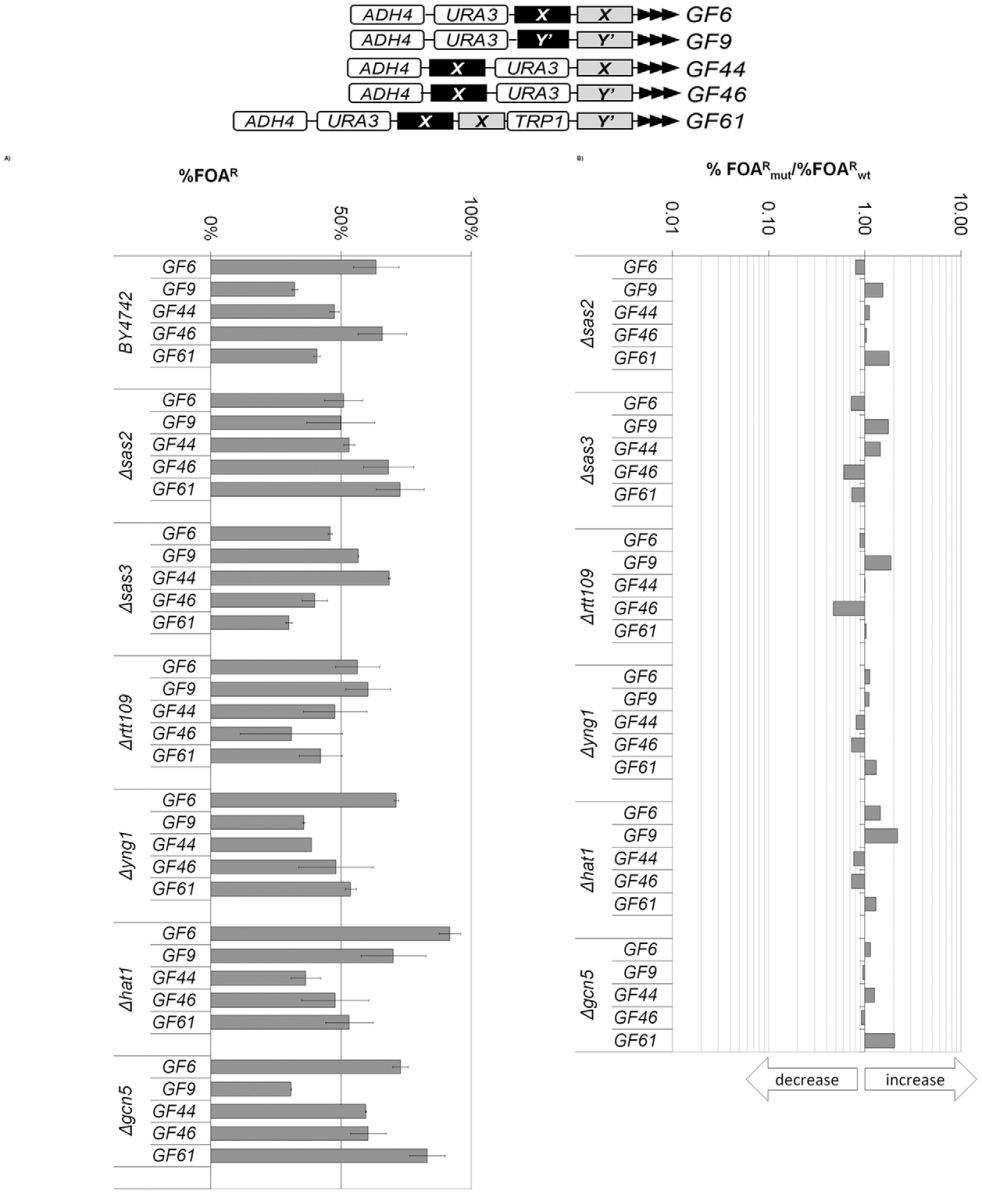
*Core X* or *Y'* restrain alterations in TPE. The GF6, GF9, GF44, GF46 and GF61 constructs (shown on top) were integrated in the strains shown on the left and the level of *URA3* silencing was calculated as %FOA^R^ cells. **A) Levels of** ***URA3*** **silencing (%FOA^R^).** **B) Ratios of %FOA^R^ in the mutant strains versus the %FOA^R^ in the** ***wild type*** **(** ***BY4742*** **) strain.** Data is from Table S1. The arrows underneath the exponential graph indicate increase or decrease of silencing.

The calculations of %FOA^R^ in the mutant strains versus %FOA^R^ in the *wild type* strain in GF6, GF9, GF44, GF46 and GF61 revealed that the silencing of these reporters was marginally influenced by the deletions of individual HAT genes (Fig. 3B). All these reporters contain a single copy of *core X* or *Y'* (black rectangles in the graphs shown on top of Figure 3). Hence, the strong repression or anti-repression effects, which were observed in URA3-tel, GF2 and GF3 (Fig. 2B) were dramatically reduced by the addition of *core X* or *Y'* regardless of the position of these elements relative to *URA3* and the telomere. The consistent decrease of silencing abbearations in all mutants and constructs strongly suggests that the subtelomeric *core X* and *Y'* curtail variations in TPE and maintain the epigenetic plasticity of these loci.

### *Core X* and *Y'* curtail variations in TPE in *cdc6-1* and *Δrif1* cells

**Figure 4:**
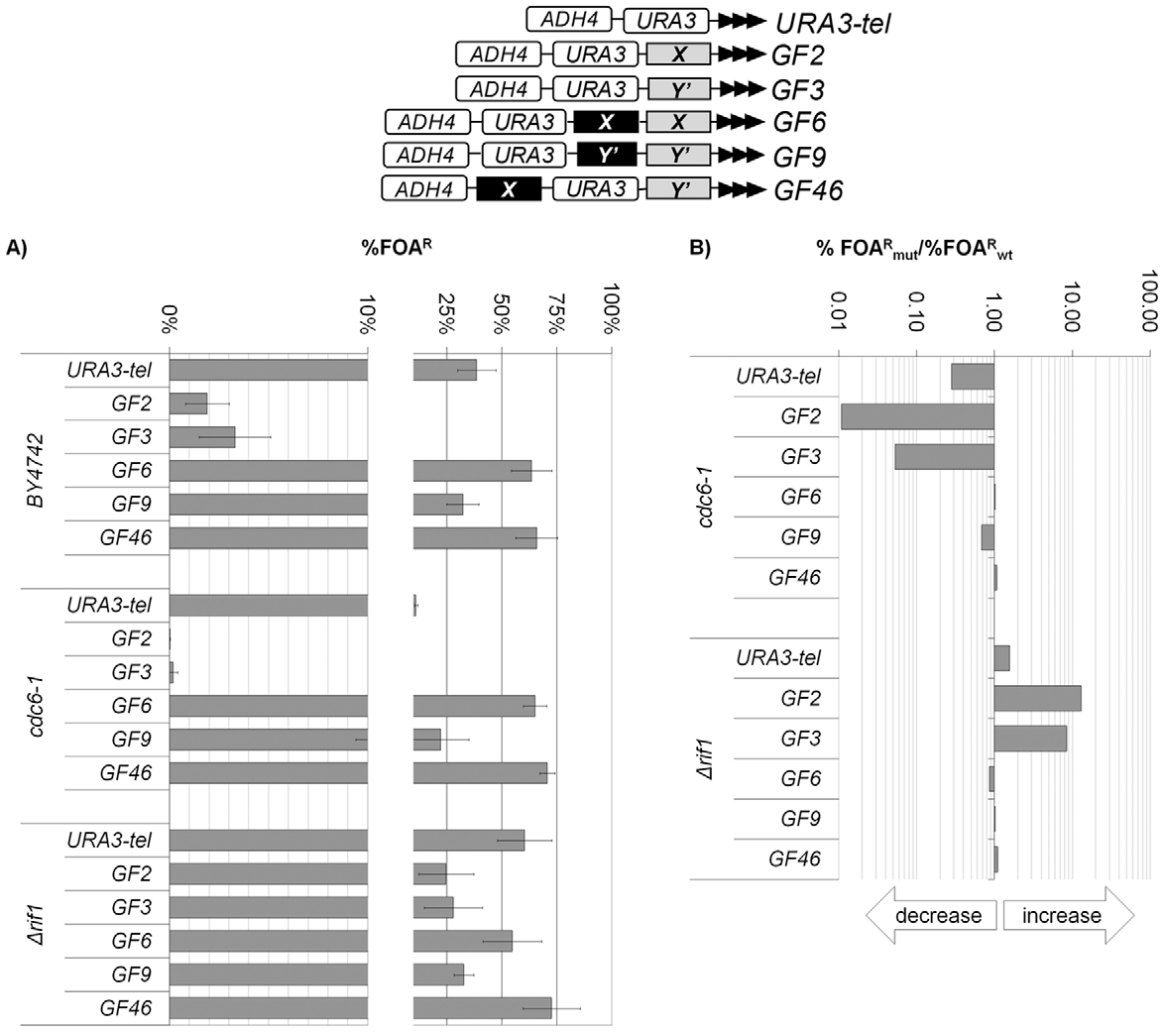
Effects of *Core X* and *Y'* in *Δrif1* and *cdc6-1* cells. The URA3-tel, GF2, GF3, GF6, GF9 and GF46 constructs (shown on top) were integrated in the strains shown on the left and the level of *URA3* silencing was calculated as %FOA^R^ cells. **A) Levels of** ***URA3*** **silencing (%FOA^R^).** The 0-10% range is spread out to properly show differences at very low levels of silencing. **B) Ratios of %FOA^R^ in the mutant strains versus the %FOA^R^ in the** ***wild type*** **strain.** *Wild type* depicts *BY474* for *Δrif1* and *W303* (not shown) for *cdc6-1*. The arrows underneath the exponential graph indicate increase or decrease of silencing.

We tested if the observed “cushioning” behavior of *X* and *Y'* is similar in non-HAT mutants. For these analyses we selected *cdc6-1* and *Δrif1* cells. Rif1p counteracts the association of Sir3p/4p with the telomere-bound Rap1p [9], [11]. Consequently, the deletion of *RIF1* boosts telomeric silencing [31]. On the other hand, the *cdc6-1* mutation dramatically reduces telomeric silencing independently of the *ACS* proto-silencers positioned in the *core X* and *Y'* elements [24]. Hence, these two mutations provide two opposing effects on TPE that are not directly mediated by *core X* and *Y'*. In Fig. 4B we show the analysis of telomeric silencing in these two mutants. As expected, *cdc6-1* and *Δrif1* significantly decreased or increased the silencing of *URA3* in the constructs lacking *core X* and *Y'* (URA3-tel, GF2, GF3). These effects were not seen in the constructs with *core X* and *Y'* (GF6, GF9, GF46). In conclusions, we observed that *core X* and *Y'* can curtail both positive and negative effects on TPE in diverse mutants.

### *ACS* and *STAR* confer opposing activities upon deletion of *GCN5* and *Rtt109*

**Figure 5:**
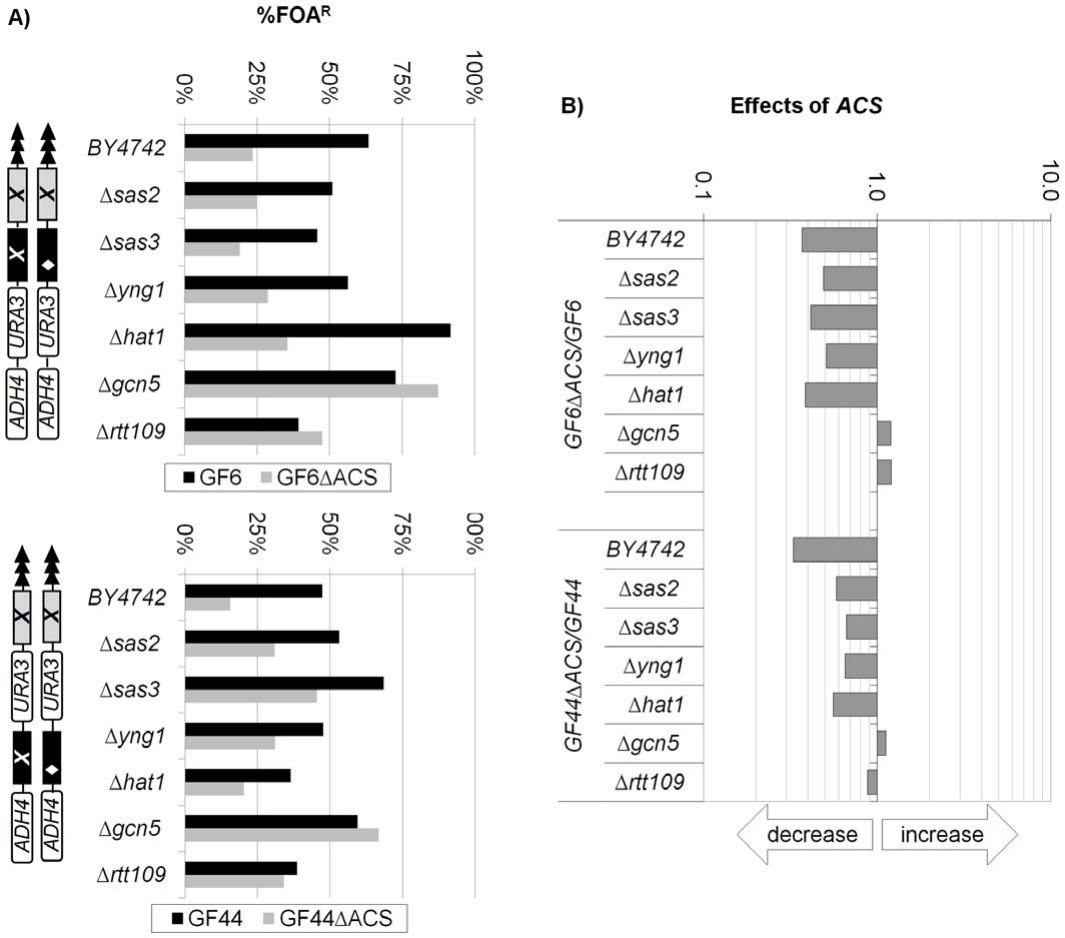
Effects of *ACS* proto-silencers in HAT deletion mutants. GF6, GF6ΔACS, GF44 and GF6ΔACS constructs (shown on the left) were integrated in the strains shown along the vertical axis and the level of *URA3* silencing was calculated as %FOA^R^ cells. **A) Levels of** ***URA3*** **silencing (%FOA^R^).** The levels of silencing of *ACS*-containing (black bars) and *ACS*-less (grey bars) constructs are shown side by side. **B) Ratios of %FOA^R^ in** ***ACS*** **-less versus** ***ACS*** **-containing constructs.** The ratios %FOA^R^_GF6ΔACS_/%FOA^R^_GF6_ and %FOA^R^_GF44ΔACS_/%FOA^R^_GF44_ were calculated and plotted. The arrows underneath the exponential graph indicate increase or decrease of silencing.

Subtelomeric *ACS* function as weak silencers [2], which relay the silencing signals emitted by the telomere. Recently we have demonstrated that in several strains, which harbor mutations in replication factor genes, *ACS* convert to weak anti-silencers [25]. Is it then possible that the cushioning effect of *core X* and *Y'* is linked to similar conversions of these ACS? We tested this possibility by destroying the *ACS* in two of the constructs to produce GF6ΔACS and GF44ΔACS. We introduced these constructs in HAT-deletion mutants and then calculated the ratios %FOA^R^_GF6ΔACS_/%FOA^R^_GF6_ and %FOA^R^_GF44ΔACS_/%FOA^R^_GF44_. The results are shown in Figure 5. The deletion of *ACS* in both GF6 and GF44 reduced the silencing in *BY4742, Δsas2, Δsas3, Δyng1* and *Δhat1* cells. In contrast, the destruction of *ACS* had very little effect in *Δgcn5* and *Δrtt109* cells. This observation suggests that *GCN5* and *Rtt109* directly or indirectly stimulate the silencing activity of subtelomeric *ACS*. At this point we can not explain the mechanism of their action. We also noticed that the deletions of *SAS2, SAS3, YNG1* and *HAT1* did not alter the *ACS*-dependent silencing in GF6 relative to *wild type* cells, while in GF44 there was about two-fold reduction in these mutants. The differences between GF6 and GF44 are obviously caused by the different position of *core X*, but at present we cannot explain the nature of this specific effect.

**Figure 6:**
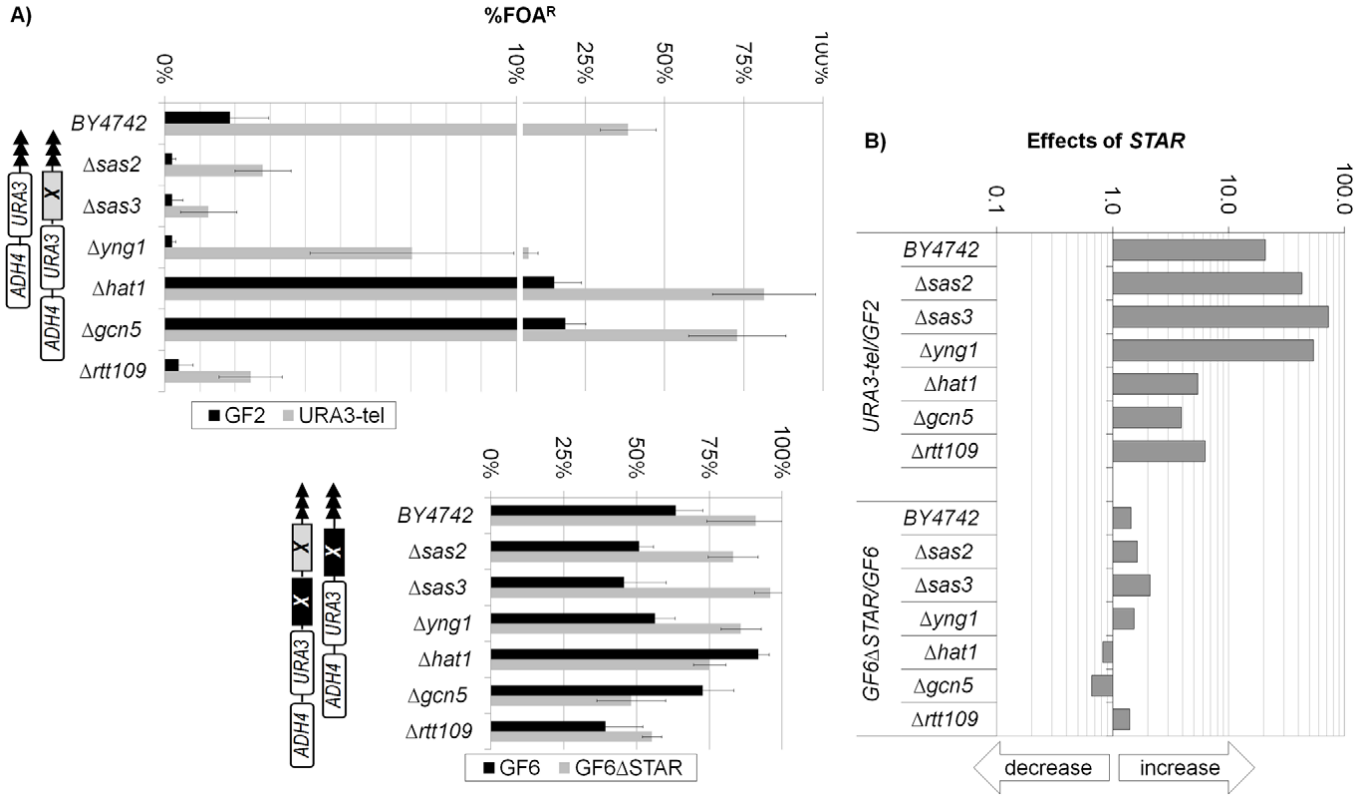
Effects of *STAR* in HAT deletion mutants. The URA3-tel, GF2, GF6 and GF6ΔACS constructs (shown on top) were integrated in the strains shown along the vertical axis and the level of *URA3* silencing was calculated as %FOA^R^ cells. **A) Levels of** ***URA3*** **silencing (%FOA^R^).** The 0-10% range in the upper graph is spread out to properly show differences at very low levels of silencing. The levels of silencing of *STAR*-containing (grey bars) and *STAR*-less (black bars) constructs are shown side by side. **B) Ratios of %FOA^R^ in** ***STAR*** **-less versus** ***STAR*** **-containing constructs.** The ratios %FOA^R^_URA3-tel_/%FOA^R^_GF2_ and %FOA^R^_GF6ΔSTAR_/%FOA^R^_GF6_ were calculated and plotted. The arrows underneath the exponential graph indicate increase or decrease of silencing.

Another set of experiments was conducted to directly assess the effects of *STARs* within the mutant strains by comparing the levels of silencing in *STAR*-less (URA3-tel and GF6ΔSTAR) and *STAR* containing (GF2 and GF6) constructs. Our calculations showed that the *STAR* in GF2 was 2-3 fold more efficient in *Δsas2*, *Δsas3* and *Δyng1* cells relative to *wild type* cells, but 4-6 fold less efficient in *Δrtt109*, *Δhat1* and *Δgcn5* cells. The *STAR* in the *core X*-containing GF6 operates at marginal efficiency. These observations demonstrated that *core X* can dominantly suppress the contribution of *STARs* to the overall level of gene silencing and that *STARs* probably function through the joint activity of *Rtt109, HAT1* and *GCN5*. More importantly, the deletions of *Rtt109* and *GCN5,* which have reduced the anti-silencing activity of the tested *STAR* (Fig. 6B) have also reduced the silencing activity of the *ACSs* proto-silencers in *core X* (Fig. 5B). These observations provide a plausible mechanism for the chromatin modulating activity of *core X* and *Y'*.

## Discussion

The comparison of eight recombinant telomeres in eight mutant strains has clearly demonstrated that *core X* and *Y'* elements curtail extreme changes in TPE. We show that telomeres without *core X* and *Y'* elements are subject to significant shifts towards de-repression or repression upon deletion of HAT genes (Figures 2, 4). In contrast, TPE remains largely undisturbed in *core X-* and *Y'*- containing telomeres (Figures 3, 4). In an earlier study we have also observed that the anti-silencing caused by mutations in DNA replication factors is also reduced by *core X-* and *Y'* [24]. Whereas the precise mechanism of the effects of each individual HAT or replication factor mutation remains unknown, it is apparent that *core X-* and *Y'* moderate all these effects. We also need to point out that the synthetic *core X-* and *Y'-* containing telomeres display moderate deviations in TPE that compare in magnitude the effects observed at natural telomeres [13], [14], [15], [16], [17].

It has been previously shown that *core X* and *Y'* contribute to gene repression, and that subtelomeres contain anti-silencing modules such as the *STARs* [3], [4], [24], [25]. The opposing signals emitted by these elements have been implicated in the variegated nature of subtelomeric gene expression [7]. An important feature of TPE at individual telomeres is that despite the seemingly random conversion between active and repressed state, the proportion of cells with active/repressed genes remains stable. The mechanisms that sustain this meta-stable balance are not so well understood. Here we propose the subtelomeric *core X* and *Y'* could play a significant and unexpected role in the dynamic meta-stability of telomeric gene expression. Previous studies have provided extensive evidence in support of their ability to reconstitute telomeric gene repression when silencing is decreased [2], [4], [5], [26]. For this reason, *core X* and *Y'* are generally viewed as proto-silencers. Our data show that these elements can also reduce telomeric gene repression when silencing increases.

We propose that these elements contain not only individual proto-silencers such as *ACS* and binding sites Rap1p and Abf1p [2], but also some unidentified anti-silencers. These anti-silencers are independent of the previously characterized *STARs*. Ultimately, the multiplicity of individual weak proto-silencers and anti-silencers in *core X* and *Y'44* build up “buffering” *cis*-elements, which suppress extreme variations in TPE. Such individual elements can acquire opposing activities upon changes of environment or in different genetic contexts. Indeed, we show that the deletion of *GCN5* or *Rtt109* reduces both the anti-silencing activity of a *STAR* and the silencing activity of an *ACS* (Fig. 4). Consequently, the net effect of the deletions of these two genes on the tested *core X-* and *Y'*- containing telomeres is minimal.

### What are the *STAR*s?

*STARs* have been characterized as anti-silencing modules residing in proximity of *core X* and *Y'* elements [4]. Independently of the *core X* and *Y'*, *STARs* reduce silencing when introduced in a modified *HMR* mating type locus [4]. The mechanism of action of *STARs* is largely unknown. They contain binding sites for Tbf1p and Reb1p thus implicating these two proteins in *STAR* activity [4], [32], but additional details are missing. Here show that *GCN5*, *RTT109* and *HAT1* affect the strength of *STAR* activity (Fig. 6B). It is therefore possible that Tbf1p and Reb1p promote the activity of these HATs. Finally, *STARs* significantly reduce the silencing only at telomeres, which do not contain *core X* or *Y'* (Fig. 4). Hence, *core X* and *Y'* activity seems dominant relative to *STARs*.

### Technical issues in studies on TPE

Several earlier studies have pointed out significant discrepancies in the silencing at natural telomeres and at synthetic telomeres on truncated chromosomes. For example, the deletion of *SAS2* had caused 10-50 fold reduction of silencing of the simple truncated URA3-tel reporter [21], [29]. Yet, RT-PCR or microarray analyses of natural subtelomeric genes had shown very moderate (two fold) alteration in expression in *Δsas2* cells [14], [15], [33].

In this study we show that synthetic telomeres, which contain *core X* and *Y'* elements, closely recapitulate the modest effects of the deletion of *SAS2* at natural telomeres. The same moderate effects apply for all other HATs tested. Hence, analyses of telomeric reporters, which contain *core X/Y'* elements, present a solid alternative to the analyses at natural telomeres.

On the other hand, “complex” synthetic telomeres can muffle weak effects on TPE. For example, studies on *SAS3* have been said to be hampered by the lack of readily detectable phenotypes [34]. Here we demonstrate a readily detectable effect of the deletion of *SAS3.* Indeed, the deletion of *SAS3* reduces telomeric silencing as strongly as the deletion of *SAS2* (Fig. 2). Therefore, “simple” synthetic telomeres need to be used for the analysis of weak silencing effects.

### Role of different HATs in TPE

This study has been initiated as a screen for the effects of different HATs on TPE before it has refocused on the consistent effects of *core X* and *Y'*. Consequently, we provide abundant data on the effects of HAT deletions on TPE. Whereas none of these effects is guaranteed to be direct, two points of potential significance need to be raised.

The first point is the modest but consistent reduction in the efficiency of *STARs* in *Δrtt109*, *Δhat1* and *Δgcn5* (Fig. 4). As mentioned, very little is known about the mode of operation of these *cis*-elements. It is premature to suggest that *STARs* recruit these HATs. The weak effects of *Rtt109*, *HAT1* and *GCN5* corroborate this notion. It is more likely that these subtelomeric regions somehow confer access to HATs, which can passively act to disrupt the spreading of heterochromatin. This hypothesis should be tested by focused mechanistic studies in single and double mutants in these genes.

The other point of discussion is the similarity in the effects of *SAS2*, *SAS3*, *YNG1* and *Rtt109* on simple telomeres. *SAS2* counteracts the deacetylation of H4-K16 by Sir2p [13], [14], [15], [16]. Hence, in these meticulous studies *SAS2* is acting as an anti-silencing factor. However, at simple telomeres or modified mating type loci the deletion of *SAS2* causes dramatic loss of repression therefore portraying *SAS2* as a silencing factor (Fig. 2 and [18], [19], [20], [21]). It is possible that loss of boundary activity and/or the redistribution of a limiting silencing factor such as Sir3p [35], [36], [37] could indirectly produce these effects. If so, *SAS3* and *Rtt109* could also act to limit the indiscriminate association of silencing factors to chromatin away from the telomere as is the case with *SAS2* [14], [15]. The possible role of these HATs in boundary formation should also be considered. In summary, the present study provides clues for the possible roles of HATs in TPE. The actual mechanism of their action will be addressed in future studies.

## Supporting Information

Table S1 Levels of gene silencing in different mutants.
(PDF)
