# Supplementary material for: Sub-Telomeric core X and Y' Elements in S.cerevisiae Suppress Extreme Variations in Gene Silencing
Source: PLoS One. 2011 Mar 17;6(3):e17523. doi: 10.1371/journal.pone.0017523 (PMC3060084; doi:10.1371/journal.pone.0017523)
Supplement: Table S1 — Levels of gene silencing in different mutants. (PDF) [file pone.0017523.s001.pdf]

Table S1. Levels of gene silencing in different mutants.

Strain	Construct	%FOA^R	±STDEV
BY4742	*URA3-tel*	38.5%	±8.8% (n=3)
	GF2	1.9%	±1.1% (n=3)
	GF3	3.3%	±1.8 %(n=3)
	GF6	63.5%	±9.3% (n=3)
	GF6ΔACS	23.4%	±1.3% (n=3)
	GF6ΔSTAR	90.9%	±16.9% (n=3)
	GF9	32.2%	±7.3% (n=3)
	GF44	47.4%	±12.8%(n=3)
	GF44ΔACS	15.6%	±1.2%(n=3)
	GF46	65.8%	±9.6% (n=3)
	GF61	40.7%	±9.2% (n=5)
Δ sas2	*URA3-tel*	2.8%	±0.8%(n=3)
	GF2	0.1%	±0.1%(n=3)
	GF3	0.4%	±0.4%(n=3)
	GF6	50.9%	±4.8%(n=3)
	GF6ΔACS	25.0%	±1.2%(n=3)
	GF6ΔSTAR	83.1%	±2.1%(n=3)
	GF9	49.8%	±8.5%(n=3)
	GF44	53.1%	±8.5%(n=3)
	GF44ΔACS	31.0%	±12.2%(n=3)
	GF46	68.2%	±19.6%(n=3)
	GF61	72.7%	±8.2%(n=3)
Δ sas3	*URA3-tel*	1.2%	±0.8%(n=3)
	GF2	0.02%	±0.03%(n=3)
	GF3	0.02%	±0.01%(n=3)
	GF6	45.8%	±14.3%(n=3)
	GF6ΔACS	19.0%	±2.1%(n=3)
	GF6ΔSTAR	95.9%	±5.5%(n=3)
	GF9	56.6%	±4.1%(n=3)
	GF44	68.5%	±12.5%(n=3)
	GF44ΔACS	45.5%	±5.7%(n=3)
	GF46	39.9%	±12.9%(n=3)
	GF61	29.9%	±9.1%(n=3)

Strain	Construct	%FOA^R	±STDEV
Δ yng1	*URA3-tel*	7.0%	±2.9%(n=3)
	GF2	0.1%	±0.1%(n=3)
	GF3	0.2%	±0.2%(n=3)
	GF6	56.3%	±6.8%(n=3)
	GF6ΔACS	28.7%	±0.7%(n=3)
	GF6ΔSTAR	85.8%	±6.9%(n=3)
	GF9	60.3%	±4.7%(n=3)
	GF44	47.7%	±15.4%(n=3)
	GF44ΔACS	31.1%	±12.1%(n=3)
	GF46	30.9%	±0.8%(n=3)
	GF61	42.1%	±8.8%(n=3)
Δ hat1	*URA3-tel*	81.3%	±16.2%(n=3)
	GF2	15.1%	±8.5%(n=3)
	GF3	19.7%	±2.7%(n=3)
	GF6	91.8%	±3.7%(n=3)
	GF6ΔACS	35.4%	±2.0%(n=3)
	GF6ΔSTAR	75.1%	±5.5%(n=3)
	GF9	70.1%	±4.7%(n=3)
	GF44	36.4%	±2.0%(n=3)
	GF44ΔACS	20.3%	±2.4%(n=3)
	GF46	47.7%	±4.6%(n=3)
	GF61	53.1%	±7.9%(n=3)
Δ gcn5	*URA3-tel*	72.9%	±15.4% (n=3)
	GF2	18.6%	±6.5%(n=3)
	GF3	15.5%	±12.9%(n=3)
	GF6	72.8%	±10.6%(n=3)
	GF6ΔACS	87.5%	±12.9%(n=3)
	GF6ΔSTAR	48.1%	±11.8%(n=3)
	GF9	30.8%	±11.4%(n=3)
	GF44	59.5%	±10.9%(n=3)
	GF44ΔACS	66.7%	±3.4%(n=3)
	GF46	60.4%	±8.20%(n=3)
	GF61	83.1%	±7.2%(n=3)

Strain	Construct	%FOA^R	±STDEV
Δ rtt109	*URA3-tel*	2.4%	±0.9%(n=3)
	GF2	0.4%	±0.4%(n=3)
	GF3	0.8%	±0.5(n=3)
	GF6	39.3%	±12.8%(n=3)
	GF6ΔACS	47.5%	±1.3%(n=3)
	GF6ΔSTAR	55.3%	±3.3%(n=3)
	GF9	35.7%	±10.1(n=3)
	GF44	38.6%	±4.1%(n=3)
	GF44ΔACS	34.1%	±1.1%(n=3)
	GF46	63.4%	±13.4%(n=3)
	GF61	53.7%	±18.3%(n=3)
Δ rif1	URA3-tel	60.2%	±12.4%(n=3)
	GF2	24.6%	±12.5%(n=3)
	GF3	27.9%	±13.2%(n=3)
	GF6	54.8%	±13.4%(n=3)
	GF9	32.7%	±4.4%(n=3)
	GF46	72.6%	±12.9%(n=3)
	GF61	28.7%	±5.1% (n=3)
cdc6-1	URA3-tel	10.9%	±0.77%(n=3)
	GF2	0.02%	±0.02%(n=3)
	GF3	0.18%	±0.24%(n=3)
	GF6	65.1%	± 5.3%(n=3)
	GF9	22.2%	±12.8%(n=3)
	GF46	70.6%	±3.5%(n=3)
	GF61	89.2%	±2.4%(n=3)
orc2-1	*URA3-tel*	0.9%	±0.5% (n=3)
orc5-1	*URA3-tel*	5.0%	±0.8% (n=3)
mcm5-461	*URA3-tel*	10.1%	±4.5% (n=3)
sas1/cdc7-1	*URA3-tel*	1.4%	±1.0% (n=3)
cdc45-1	*URA3-tel*	2.4%	±3.3 %(n=3)

12.42%
12.58%
13.25%
13.44%
4.50%
12.99%
5.04%

0.8%
0.02%
0.24%
5.3%
12.8%
3.5%
2.4%
